# Melanoma-derived extracellular vesicles transfer proangiogenic factors

**DOI:** 10.32604/or.2024.055449

**Published:** 2025-01-16

**Authors:** MAGDALENA WILCZAK, MAGDALENA SURMAN, MAłGORZATA PRZYBYłO

**Affiliations:** 1 Department of Glycoconjugate Biochemistry, Faculty of Biology, Institute of Zoology and Biomedical Research, Jagiellonian University, Krakow, 30-387, Poland; 2 Doctoral School of Exact and Natural Sciences, Jagiellonian University, Krakow, 30-348, Poland

**Keywords:** Angiogenesis, Exosomes, Extracellular Vesicles (EVs), Melanoma, Microvesicles

## Abstract

Angiogenesis, the expansion of pre-existing vascular networks, is crucial for normal organ growth and tissue repair, but is also involved in various pathologies, including inflammation, ischemia, diabetes, and cancer. In solid tumors, angiogenesis supports growth, nutrient delivery, waste removal, and metastasis. Tumors can induce angiogenesis through proangiogenic factors including VEGF, FGF-2, PDGF, angiopoietins, HGF, TNF, IL-6, SCF, tryptase, and chymase. This balance is disrupted in tumors, and extracellular vesicles (EVs) contribute to this by transferring proangiogenic factors and increasing their expression in endothelial cells (ECs). Malignant melanoma, a particular type of skin cancer, accounts for only 1% of skin cancer cases but more than 75% of deaths. Its incidence has risen significantly, with a 40% increase between 2012 and 2022, especially in fair-skinned populations. Advanced metastatic stages have a high mortality due to delayed diagnosis. This review examines the molecular basis of angiogenesis in melanoma, focusing on melanoma-derived EVs and their possible use in new antiangiogenic therapies.

## Introduction

Angiogenesis is a multistep process that leads to the expansion of pre-existing vascular and microvascular networks in all organs and tissues [1]. It is essential for normal organ growth and is required for the repair of any damaged tissue. However, aberrant angiogenesis can be observed in a variety of pathologies, including inflammation, ischemia, diabetes, varicose veins, hemangiomas, aneurysms, and many others. Angiogenesis plays a particularly important role in cancer, as solid tumors larger than a few millimeters in size require a constant blood supply. Tumor vasculature enables oxygen and nutrient delivery to growing tumors, removal of metabolic wastes, and provides a route for local and distant metastasis [1].

Most tumors are also capable of producing various molecular signals to induce or enhance angiogenesis. Main proangiogenic factors can be divided into two groups, i.e., 1) classical factors such as angiopoietins (ANGPTs), fibroblast growth factor-2 (FGF-2), hepatocyte growth factor (HGF), interleukin-6 (IL-6), platelet-derived growth factor (PDGF), tumor necrosis factor (TNF), and vascular endothelial growth factor (VEGF), as well as 2) non-classical factors such as chymase, stem cell factor (SCF), and tryptase [2–4]. The above-mentioned and other proangiogenic factors act in balance with antiangiogenic molecules under physiological conditions.

Disruption of this homeostasis is observed in the tumor microenvironment (TME). The secretion and intercellular transfer of proangiogenic factors are increased, causing alternations to the structure of the extracellular matrix (ECM) and expansion of tumor vasculature. The factors contributing to tumor angiogenesis include among others extracellular vesicles (EVs)–nanosized, phospholipid bilayer-enclosed particles, involved in the horizontal transfer of specific molecular cargo between almost all cell types in the human body. EVs have already been shown to carry several proangiogenic factors, including VEGF, matrix metalloproteinases (MMPs), and their endogenous activator CD147, PDGF, microRNAs, and lncRNAs as well as up-regulating their expression in ECs [5,6], thus facilitating interactions between tumor and ECs or other cells involved in angiogenesis.

Malignant melanoma is one of the most aggressive cancers, and although it is responsible for only 1% of skin cancer cases, it accounts for more than 75% of skin cancer-related mortality [7,8]. The last few decades have brought a decline in the incidence and mortality rates of most cancers, but nevertheless, the incidence of melanoma has increased, especially in fair-skinned populations. Between 2012 and 2022, there was more than 40% increase in melanoma cases per year [9]. A particularly high mortality rate is associated with advanced metastatic stages of the disease (Stages III and IV), mainly due to delayed diagnosis. Intravasation of tumor cells is a key step to subsequent metastasis and disease containment at this stage significantly improves patient outcomes.

Therefore, in this review, we cover the molecular basis behind angiogenesis in melanoma. We focus on the role of melanoma-derived EVs in this process and on their possible use in new antiangiogenic therapies.

## Angiogenesis in Melanoma

Angiogenesis is central to the progression of solid tumors, and melanoma is no exception. The development of primary cutaneous melanoma is usually divided into two growth phases, radial phase and vertical phase. In the radial phase, melanoma forms an irregular plaque, and tumor cells cannot invade the dermis. In the vertical phase, the lesion grows vertically into deeper parts of the dermis. During the vertical growth phase, angiogenesis is crucial for tumor growth and metastasis, which is strictly associated with the invasion of blood or lymphatic vessels. Melanoma metastatic sites are most commonly observed in the bones, lungs, liver, and brain [10].

### Mechanism of angiogenesis

**Figure 1:**
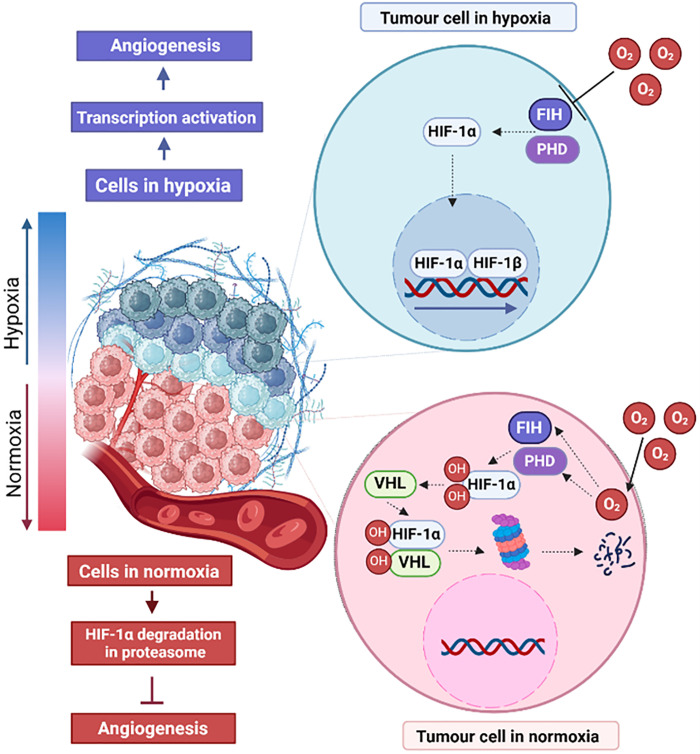
Effects of normoxia and hypoxia on angiogenesis in tumor cells. HIF-1α-hypoxia-inducible factor 1 subunit α, HIF-1β-hypoxia-inducible factor 1 subunit β, VHL-Von Hippel–Lindau tumor suppressor, PHD-prolyl hydroxylase domain enzyme, FIH-factor-inhibiting HIF. The figure was prepared under the license in BioRender: Scientific Image and Illustration Software.

The uncontrolled proliferation of melanoma cells leads to increased energy demand, which manifests as a drastic decrease in oxygen in TME and the development of hypoxia. Hypoxia is present in 90% of solid tumors and occurs in tissues more than 100–200 µM away from the functional blood supply [11]. In response to hypoxia the hypoxia-inducible factor (HIF) pathway is activated (Fig. 1). HIF is composed of an alpha subunit (HIF-α) and a beta subunit (HIF-β). HIF-α is the main transcription regulator of the developmental response to hypoxia [12]. The HIF-α subunits (HIF-1α, HIF-2α, and HIF-3α) are stabilized by enzymes that are oxygen sensors, i.e., factor inhibiting HIF-1 (FIH-1) and prolyl hydroxylase domain (PHD) [13,14]. In an oxygen-available environment, PHD and FIH-1 hydroxylate HIF-α, and hydroxylated HIF-α are then tagged by Von Hippel–Lindau tumor suppressor with E3 ubiquitin ligase activity. Ubiquitinated HIF-α is then degraded in proteasomes [15]. Reduced oxygen availability leads to inhibition of PHD and FIH-1 function and stops degradation of HIF-α subunits, which are transported to the nucleus and dimerized with HIF-β subunits and act as a transcription factor [16].

The HIF-1α and the nuclear factor ĸB (NF-ĸB) pathways interact with other proangiogenic factors, such as cyclooxygenase-2 (COX-2), cytokine-inducible nitric oxide synthase (iNOS), and stromal cell-derived factor 1(SDF-1) VEGF, and VEGF receptor (VEGFR) [17]. HIF-α is mainly involved in the recruitment of bone marrow endothelial progenitor cells (EPCs), which later differentiate into ECs via the VEGF pathway, thereby stimulating vascularization [16].

Angiogenesis is preceded by vasculogenesis, *de novo* formation of a primitive vascular network (mainly during embryogenesis) from bone marrow-derived EPCs. Subsequently, this pre-existing vasculature, made of differentiated ECs, takes part in the formation and expansion of new blood vessels in angiogenesis. There are several mechanisms of blood vessel formation [18–20]. The vessels formed by ECs are then strengthened by pericytes and smooth muscle cells, which enable perfusion. Blood vessels can grow both by sprouting and by a non-sprouting mechanism of intussusceptive microvascular growth (IMG). In sprouting angiogenesis, the basement membrane undergoes rearrangement at the site of the dilated peritumoral postcapillary venule in proximity to the angiogenic stimulus. The ECs then relocate to the connective tissue to form a solid cord, and the migrating front leads to lumen formation. Non-sprouting angiogenesis is a recovery adaptation of the existing microvascular network. Unlike traditional angiogenesis, which depends on the rapid proliferation of ECs, this process results from the reorganization of existing ECs and the incorporation of EPCs. Intussusceptive angiogenesis is related to the presence of the intussusceptive pillar, a transvascular tissue bridge of 1–5 μm in length that spans the vessel lumen. Therefore, the vascular network expands by the insertion of pillars.

### Proangiogenic and antiangiogenic factors in melanoma

**Figure 2:**
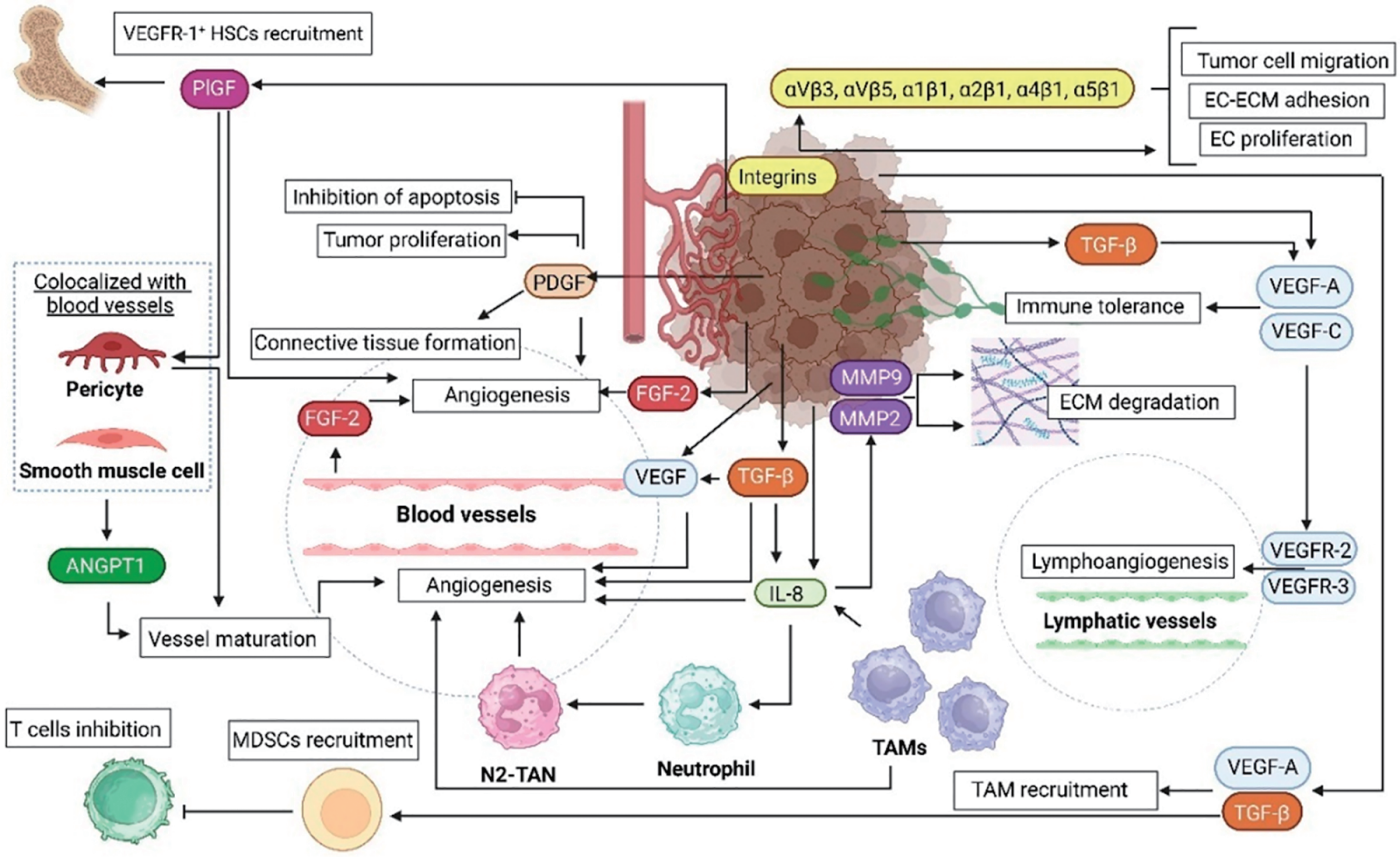
Schematic summary of the role of proangiogenic factors in melanoma. Melanoma microenvironment consists of different cell types, such as immune system cells (TAMs, neutrophils, T cells) and endothelial cells. These cells cooperate in multiple processes involved in melanoma progression, one of which is angiogenesis. The coexistence of the cells forms paracrine loops involved in ECM degradation, lymphoangiogenesis, and importantly the formation of new blood vessel and their maturation. ANGPT–angiopoietin, EC–endothelial cell, ECM–extracellular matrix, FGF–fibroblast growth factor, HSC–hematopoietic stem cell, IL-8–interleukin 8, MDSC–myeloid-derived suppressor cell, MMP–matrix metalloproteinase, PDGF–platelet-derived growth factor, PlGF–placental growth factor, TAM–tumor-associated macrophages, TGF-β–transforming growth factor β, VEGF(R)–vascular endothelial growth factor (receptor). The figure was prepared under the license in BioRender: Scientific Image and Illustration Software.

There are many proangiogenic factors released by melanoma cells (Fig. 2), which form a complex intra-and intercellular signaling network [4]. Some of these factors including VEGF, PDGF, or ANGPTs, generally stimulate melanoma-related angiogenesis by directly affecting the basic functions (such as proliferation or migration) of ECs. In contrast, factors such as IL-8, metalloproteinases (MMPs), or integrins are more indirect mediators, although still important contributors to the development of tumor vasculature. They usually act as cofactors for the factors from the first group or promote their secretion. They may be also involved in ECM remodeling required for angiogenesis, without direct interaction with ECs [4]. The specific mechanisms of action of the main proangiogenic factor identified in melanoma are described in the following subsections.

#### Vascular endothelial growth factor (VEGF)

VEGF is a signaling protein produced by various types of cells. The human VEGF family consists of VEGF-A, B, C, D, and E. All VEGF isoforms elicit a response through cell surface tyrosine kinase receptors. Binding of VEGF results in their dimerization and activation by transphosphorylation [21]. VEGF has been shown to be the survival factor for ECs *in vivo* and *in vitro* [22]. ECs of newly formed vessels functionally rely on VEGF, in contrast to established vessels within tumors. The loss of VEGF dependence may be explained by the coverage of ECs by pericytes [23].

VEGF also contributes to the horizontal/vertical growth phase transition in melanoma [24]. Moreover, strong reactivity in anti-VEGF immunohistochemical staining of melanoma specimens and increased microvascular density correlate positively with the formation of tumors of larger thicknesses (>3.6 mm). This supports the hypothesis that VEGF is involved in increasing vessel diameter and IMG in melanoma [25].

Studies on melanoma cell lines also revealed that cells with low VEGF expression can be stimulated to increase VEGF secretion by culture in hypoxic conditions [26]. Clinical studies have also shown a positive correlation between VEGF levels and Breslow scale depth and with Clark scale levels [27].

Furthermore, VEGF-A promotes the proliferation of VEGFR-2-positive cells in lymphatic vessels and metastasis to sentinel and distant lymph nodes [28]. VEGF-C also induces tumor lymphangiogenesis and enhances melanoma metastasis to lymph nodes by binding to VEGFR-3 [29]. VEGF-C expression, as well as intratumoral lymphatic vessel density (LVD), peritumoral LVD, melanoma thickness, and Clark level are good predictors of lymph node metastasis in melanoma [30]. Similarly, VEGF-C/D expression in melanoma lymph node metastases was higher than in non-metastatic melanomas [31]. VEGF-C also enhances the transport of tumor cells to the draining lymph node and their exposure to immune cells. In murine melanoma, VEGF-C promoted immune tolerance [32]. Finally, VEGF-B was found to inhibit angiogenesis. The mechanism of action was based on the FGF-B binding to FGFR1, and subsequent FGFR1/VEGFR1 complex formation, which blocked Erk activation by FGF-B [33].

#### Platelet-derived growth factor (PDGF)

PDGF glycoprotein mainly exists in three variants, i.e., PDGF-AA consisting of two A subunits, PDGF-BB consisting of two B subunits, and PDGF-AB heterodimer. In the early 2000s, two additional family members, PDGF-C and PDGF-D, were identified as novel ligands for PDGF receptors (PDGFRs) [34]. Primary and metastatic melanoma is characterized by overexpression of two PDGFRs, namely PDGFR-α and PDGFR-β, compared to normal skin [35]. PDGF is secreted by melanoma cell lines and tumors, stimulating the development of new blood vessels [35]. Moreover, it inhibits melanoma apoptosis, promotes cell cycle progression enhances melanoma cell survival [36], and exhibits mitogenic properties for melanoma cells by stimulating their proliferation through MAPK/ERK and PI3K/Akt pathways.

Mice inoculated with PDGF-BB-transfected B16 melanoma cells showed an increased pericyte coverage of tumor blood vessels [37]. However, susceptibility-contrast Magnetic Resonance Imaging (MRI) showed a significant reduction in the vessel size index in tumors formed by B16 cells with PDGF overexpression [37]. Similarly, PDGFR-α overexpression abrogated the growth of some melanoma tumors [38]. Additionally, tumor-derived PDGF-BB dimer may mediate connective tissue stroma formation as mice inoculated with WM9 melanoma cells lacking PDGF-B subunit expression showed highly necrotic tumors with narrow lumen blood vessels and lack of connective tissue. Tumors formed by WM9 cells with PDGF-B subunit overexpression had properly developed vessels and connective tissue, which resulted in a lack of necrosis [39].

#### Interleukin-8 (IL-8)

Human IL-8 is currently called chemokine (C-X-C motif) ligand 8 (CXCL8), and its receptors, previously known as IL8Rα and IL8Rβ, are now named CXCR1 and CXCR2, respectively [40]. In melanoma, CXCR2 expression is increased compared to neutrophils [41]. This results in increased binding of IL-8 to the tumor instead of immune cells. Normal melanocytes do not produce IL-8 unless stimulated. In melanoma, IL-8 mRNA is consistently expressed and is correlated with the tumor’s ability to metastasize [42]. However, IL-8 mRNA has also been observed in non-metastatic melanoma [38].

IL-8 is released by macrophages, epithelial or ECs and attracts neutrophils in infections and injuries. This results not only in the removal of pathogens, but also in enhanced angiogenesis, and the synthesis of MMPs [43,44]. Dermal microvascular ECs express IL8Rs, so IL-8 regulates MMP-2/-9 secretion and subsequent angiogenesis [45]. IL-8 is also a target of the nuclear factor of activated T cells, i.e., NFAT1 and NFATC2. NFATs are involved in immune response, but also in melanoma progression and metastasis. NFAT1 binds to the IL-8 promoter and increases IL-8 transcription, thereby promoting tumor development, growth, and dissemination [46].

Tumor-associated neutrophils (TANs) have anti-or pro-tumor phenotypes, N1-TANs, and N2-TANs, respectively [47]. IL-8 is one of the major factors in the N1-/N2-TANs balance, suppressing antitumor immunity by recruiting N2-TANs. TANs can produce IL-8, further stimulating neutrophil migration, vessel formation, and tumor growth [48]. Also, tumor-associated macrophages (TAMs) are key effectors in tumor angiogenesis, especially macrophage-derived angiogenesis and tumor invasiveness through processes mediated by IL-8 [49].

#### Fibroblast growth factor-2 (FGF-2)

FGF-2, also known as the basic fibroblast growth factor (bFGF), is a cytokine that binds to the fibroblast growth factor receptor (FGFR) [50,51]. FGF-2 is produced by melanoma cells and ECs [52]. In tumors, FGF-2 through FGFR2 stimulates the proliferation of pericytes and activates PDGFRβ signaling required for pericyte migration to the angiogenesis site [53]. During angiogenesis in melanoma, FGF-2 cooperates with heparinase, which enzymatically cleaves the glycosaminoglycan chains of heparan sulfate proteoglycans, which act as a receptor or coreceptor for FGFR [54].

Abnormal expression of FGF-2 (along with FGF-18) and elevated FGFR1 and FGFR3 characterize primary melanoma *vs*. healthy skin [55]. Also, analyses of the FGFR4 Arg388 polymorphism in 185 melanoma patients identified Arg388 allele in 45% of patients and was associated with tumor size and high microvascular density [56].

#### Angiopoietins (ANGPTs)

Four ANGPTs are known: ANGPT1, ANGPT2, ANGPTL3, and ANGPT4 [57]. ANGPT1 is secreted by pericytes and vascular smooth muscle cells. It is a key factor in the blood vessel maturation, adhesion, and migration of ECs. Its proangiogenic activity is induced by binding to the angiopoietin-1 receptor (Tie-2), which increases vessel quiescence and inhibits vascular permeability. ANGPT1 is significantly overexpressed in the vasculature of most tumors, and in ANGPT-1-deficient mice, MT-ret and B16F10 melanomas grow more slowly [58]. ANGPT2 is released by ECs and may act as a Tie-2 antagonist, leading to temporary disruption of existing blood vessels required for their further development, i.e., angiogenesis [57,59]. The balance between ANGPT1 and ANGPT2 undergoes a proangiogenic shift in melanoma. High levels of ANGPT2 compared to ANGPT1 positively correlate with tumor vascularity, tumor growth, and poorer prognosis [60].

#### Transforming growth factor β (TGF-β)

TGF-β is a class of cytokines with multiple biological functions. The TGF-β family includes a variety of molecular subtypes, among which TGF-β1, -β2, and -β3 are most extensively studied to date. Unlike normal melanocytes, melanoma cells not only escape cell cycle arrest induced by TGF-β but also produce it and respond to it at the gene level [61]. TGF-β1 is produced by melanocytes and melanoma tumor cells. On the other hand, TGF-β2 and -β3 are expressed heterogeneously only in nevi and melanomas, and their expression increases during tumor progression [62].

TGF-β is involved in the formation of peri-tumoral blood vessels by stimulating the secretion of IL-8 and VEGF-A. TGF-β also activates the migration of ECs to sites of angiogenesis within the tumor niche [61]. Endoglin (CD105) is a TGF-β receptor binding TGF-β1 and -β3, but not TGF-β2 [63]. In ECs, endoglin is crucial for angiogenesis since it facilitates the binding of TGF-β family members to activin receptor-like Kinase 1 (ALK 1). Endoglin expression in melanoma indicates angiogenesis [64].

Moreover, two bone morphogenetic proteins (BMPs) from the TGF-β family, namely BMP-4 and BMP-7, are frequently overexpressed in melanoma. Importantly, downregulated expression of BMP-4 in melanoma cells correlated with their diminished proangiogenic paracrine activity [65,66].

#### Placental growth factor (PlGF)

Melanoma cells express two PIGF isoforms, namely PlGF-1 and PlGF-2, which bind to neuropilin-1 and neuropilin-2 receptors on ECs [67]. In addition, PlGF forms heterodimers with VEGF, indirectly interacting with VEGFR-2 on ECs [68,69]. PlGF also enhances blood vessel maturation by interacting with VEGFR-1-positive pericytes [68]. Moreover, PlGF contributed to increased vessel branching, size, and stability *in vivo* [70]. Finally, a comparison of PlGF levels between patients with metastatic melanoma and healthy control revealed 20-fold higher plasma PlGF levels in patients [71].

#### Matrix metalloproteinases (MMPs)

MMPs are a group of more than 20 Zn^2+^-dependent endogenous peptidases that participate in wound healing, tissue remodeling, and angiogenesis [72,73]. MMPs catalyze the degradation of collagen, gelatin, elastin, fibronectin, and laminin, which are key ECM components. As a consequence, their activity supports tumor metastasis, allowing tumor cell migration from primary sites to metastatic niches [74].

All MMPs expressed in melanoma cells directly or indirectly participate in angiogenesis [75,76]. IL-8 enhances the activity of MMP-2 secreted by melanoma cells, which in turn promote melanoma invasion [77]. Moreover, through interaction between membrane matrix metalloproteinase type 1, MMP-2, and laminin-5γ2 chain fragments melanoma cells tend to display vasculogenic mimicry, the phenomenon in which tumor cells mimic EC activity to participate in neovascularization and the formation of a matrix-rich meshwork [78].

In melanoma cells, MMP-9 colocalizes with CD44, this interaction promotes the proteolytic activity of MMP-9 against type IV collagen [79]. ECM degradation by MMP-9 induces secretion of FGF and VEGF [80]. Moreover, in melanoma cells, the rapamycin-insensitive companion of mTOR complex 2 (Rictor-mTORC2) can phosphorylate AKT, causing overexpression of MMP-2 and MMP-9 and microvessel formation [81]. However, MMP-9 has been shown to be expressed only during the horizontal but not vertical growth phase [82]. Malignant melanoma cells express not only MMP-2 and 9, but also MMP-1, 13, and 14, and their inhibitors such as TIMP-1, 2, and 3 [75,76].

#### Integrins

The protein family of integrins consists of heterodimeric (α and β subunits with non-covalent linkage) transmembrane cell surface receptors involved in cell-cell and cell-ECM adhesion. Integrin signaling cascades also modulate cell proliferation and survival, as shown, for example, in the focal adhesion kinase (FAK), Rho GTPase, MAPK/ERK, and PI3K/Akt/mTOR pathways. After the transition from the primary to metastatic phase melanoma cells overexpress chosen integrins, namely αvβ3, αvβ5, α2β1, α4β1 α1β1, and α5β1 integrins [60].

A major part of research concerning the proangiogenic role of integrins was related to αvβ3 integrin. Integrin αvβ3 is a classic vitronectin receptor, often overexpressed in developing blood vessels and required for angiogenesis. The β3 integrin subunit expressed by ECs can undergo phosphorylation via the VEGF/c-Src pathway, and the phosphorylated β3 integrin subunit promotes activation of VEGFR-2. Blocking αvβ3 integrin with antibodies or low-molecular-weight antagonists, (e.g., arginyl glycyl aspartic acid (RGD) mimetics) inhibited angiogenesis in *in vitro* and *in vivo* models [83,84], including tumor angiogenesis in melanoma. Knockout of αvβ3 integrin in mouse melanoma tumors led to reduced tumor growth and microvessel density [84,85]. Also, conditioned media from αvβ3 integrin-expressing and αvβ3 integrin-non-expressing melanoma cell cultures were collected and added to HUVEC cells. ECs treated with media deprived of αvβ3 integrin showed a significantly lower proliferation rate in the MTT assay, suggesting that αvβ3 integrin present in the melanoma secretome has a measurable functional effect on ECs [85]. Moreover, melanoma cells co-expressing α2bβ3 and αvβ3 integrins were shown to also have an increased expression of FGF-2 [86].

Another integrin, αvβ5 integrin, is involved in the regulation of neuropilin 1 (NRP-1)-dependent angiogenic pathways in melanoma. Inhibition of αvβ5 integrin prevents the formation of NRP-1/VEGF-A complexes and abrogates angiogenesis [82]. In addition, the β1 integrin subunit was found to be required for melanoma cell adhesion to ECs [87], mainly through the FAK/paxillin pathway, and for the extravasation of melanoma cells at metastasis sites (mainly in liver and lung) [88,89]. Also, α5β1 integrin modulates ANG-1-dependent angiogenesis through interaction with Tie-2 [90].

## Extracellular Vesicles (EVs)

**Figure 3:**
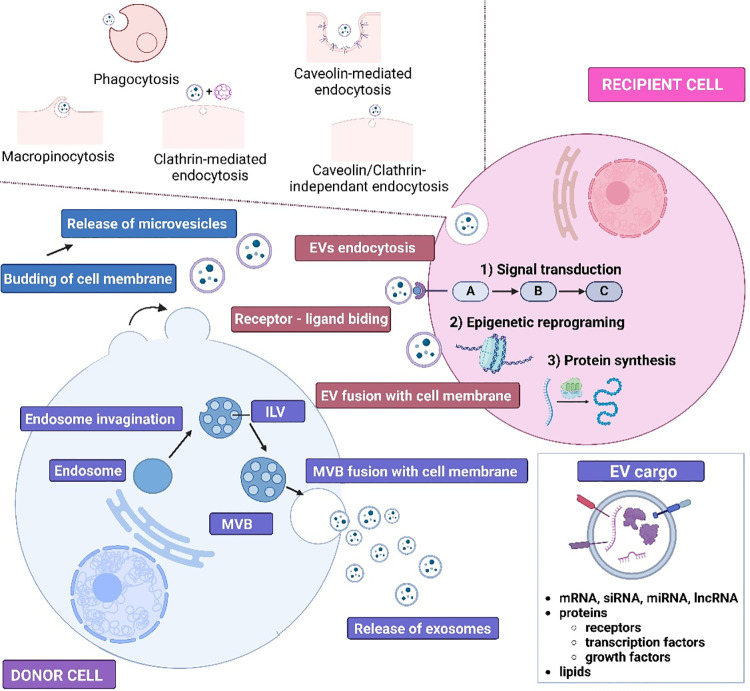
Schematic presentation of exosomes and microvesicles biogenesis and the ways of cargo transmission to recipient cells. ILV–Intraluminal vesicle, MVB–multivesicular body, EVs–extracellular vesicles, mRNA–messenger RNA, siRNA–small interfering RNA, miRNA–microRNA, lncRNA–long non-coding RNA. The figure was prepared under the license in BioRender: Scientific Image and Illustration Software.

Interest in EVs is constantly increasing due to their enormous diagnostic and therapeutic potential. EVs can be isolated from body fluids (blood, breast milk, cerebrospinal fluid, saliva, sperm, urine,), and conditioned culture media [91]. Within EVs, three subpopulations of vesicular structures are usually distinguished, i.e., exosomes, ectosomes (also known as microvesicles), and apoptotic bodies (Fig. 3). The largest EVs are apoptotic bodies (ABs) (1000 to 5000 nm in diameter), which are released by cell shrinkage during apoptotic death. Biogenesis of ectosomes involves cell membrane budding caused by rearrangements in the cytoskeleton. MVs are also released constitutively, and an increase in their production by cells is usually associated with the action of various stressors, such as nutrient deprivation or increased Ca^2+^ levels. The diameter of MVs varies between 100–1000 nm. Exosomes are the smallest EVs (30 to 100 nm in diameter). Exosome biogenesis is a multistep process, in which endosomes are involved. After invagination of the endosome membrane intraluminal vesicles (ILVs) are formed. From this point on, endosomes transporting ILVs are called multivesicular bodies (MVBs). Once MVBs fuse with the cell membrane, exosomes are released to the extracellular space [92,93].

However, the terminology for classifying EVs, including “exosomes”, “microvesicles/ectosomes” and “apoptotic bodies”, should be used with caution in accordance with Minimal information for studies of extracellular vesicles (MISEV2023) guidelines [94]. Most isolation methods (e.g., filtration or differential ultracentrifugation) separate EVs according to their size and density, whereas the terminology described here relates more strictly to their biogenesis. The size ranges (30–100 nm and 100–1000 nm) are highly arbitrary–exosomes and ectosomes probably overlap in size, especially around 100 ± 50 nm. Therefore, terms such as “small” (<200 nm) and “large” (>200 nm) have been commonly used to denote EV populations over the past few years, unless the cellular origin (endosomal *vs*. outer cell membrane) can be clearly demonstrated for an isolated sample. Thus, while different terminologies can still be used, researchers should be aware of their limitations and aim to characterize the EVs under study as clearly as possible [94].

When EVs were first observed, no role was assigned to them, as they were considered a type of cellular waste. It was later discovered that they participate in cellular communication and are involved in various physiological and pathological processes, e.g., autoimmune [95] and neurodegenerative diseases [96], processes associated with transplanted organ rejection [97], and cancer [98]. In carcinogenesis, EVs facilitate drug resistance, angiogenesis, epithelial-mesenchymal transition, invasion, migration, escape from apoptosis, and both pro-and anti-tumor stimulation of the immune system. All of these processes may be modified by the cargo transported by EVs, including metabolites, active forms of lipids, proteins (e.g., transcription factors, receptors, growth factors), and a broad panel of nucleic acids, including mRNAs, lncRNAs, and miRNAs [99].

The EV cargo largely mirrors the state of the cell that released them. The molecular information contained in EVs can be transmitted to the recipient cells in several ways as shown in Fig. 3 [100]. EVs can interact with recipient cells through various types of endocytosis, such as caveolin- or clathrin-mediated endocytosis, micropinocytosis, lipid raft-mediated endocytosis, or phagocytosis. EVs can also directly fuse with the membrane of recipient cell. Finally, the molecular signal can be transduced via receptor-ligand interaction. Therefore, the interaction between EVs and recipient cells can result in epigenetic reprogramming or inducing or inhibiting cellular pathways in recipient cells.

## Role of Melanoma-Derived EVs in Angiogenesis

Despite significant advances in EV research, knowledge of their role in melanoma-related angiogenesis is still very limited. Nevertheless, as mentioned above, the presence of various pro-and/or-antiangiogenic factors in melanoma-derived EVs has already been confirmed [5,6]. Several different mechanisms by which EVs may promote angiogenesis have also been investigated. In the following section, we review the available research data on the effect of melanoma-derived EVs on tumor angiogenesis and discuss their therapeutic and prognostic potential.

### Transfer of proangiogenic factors and regulation of their expression in endothelial cells

Melanoma-derived EVs regulate angiogenesis itself or modulate related processes such as ECM remodeling. First, the urokinase-type plasminogen activator receptor (uPAR) carried by them increased the expression of EGFR, uPAR, and VE-cadherin, and activated the ERK1/2 pathway in recipient ECs [101]. Functional EGFR can also be directly transferred from melanoma to ECs via larger EVs, i.e., ectosomes (microvesicles), and such transfer depends on the phosphatidyl serine (PS) presence on the EV surface [101]. As a result, EGFR activates MAPK/Akt pathways in recipient cells and increases the expression of VEGF and its receptor VEGFR-2, thereby activating autocrine VEGF-VEGFR-2 signaling.

Moreover, overexpression of Wnt Family Member 5A (WNT5A) in several melanoma cell lines was associated with increased release of exosomes enriched in VEGF, IL-6, IL-8, and MMP-2 [102]. In addition, exosomes derived from melanoma cells overexpressing WNTA5A enhanced tube formation by ECs on Matrigel [102]. To follow up on matrix-degrading enzymes, membrane-type 1 matrix metalloproteinase (MT1-MMP) was also identified in melanoma-derived EVs [103].

Another proangiogenic factor identified in melanoma-derived EVs is tissue factor (TF). Alongside its procoagulant activity, TF can also stimulate signaling via the PAR-2 receptor, leading to upregulation of VEGF and enhanced angiogenesis. In addition, melanoma-derived EVs have been shown to contain more TF than melanocyte-derived EVs [104].

Finally, Hood et al. described the most complex 3D *in vitro* model of endothelial tissue to better mimic the microenvironment and morphology of ECs [105]. It was found that melanoma-derived exosomes are transferred between ECs via tunneling nanotubes. Moreover, melanoma-derived exosomes stimulated the formation of sprouting endothelial spheroids and the secretion of various proangiogenic cytokines. Interestingly, the secretion of IL-1α, FGF, GCS-F, TNFα, leptin, TGF-α, and VEGF, by endothelial spheroids correlated positively with the exosome dose used for incubation. These data provided evidence supporting the involvement of exosomes in endothelial angiogenic responses.

### Induction of proangiogenic switch in bone marrow progenitor cells

Bone marrow-derived progenitor cells (BMPCs) are known to promote both, neoangiogenesis and remodeling of existing blood vessels. Peinado et al. showed that melanoma-derived exosomes induce a proangiogenic phenotype in BMPCs associated with the horizontal transfer of c-Kit, Tie-2, and Met oncoprotein [106]. Moreover, in melanoma-bearing B16F10 mice, the same exosomes enhanced the formation of pulmonary pre-metastatic niches with typical leaky vasculature that supports subsequent metastasis. Such an effect was not observed when exosomes with lower Met content were used, indicating that exosomal Met is a key factor determinant of the proangiogenic switch in BMPCs in melanoma. Similar findings were later made in another study in which the same B16F10 mice were also inoculated with Met-enriched exosomes [107]. It led to increased numbers of lung and femur metastases, while exosomes with lower Met expression did not induce significant changes.

### Generation of cancer-associated fibroblasts (CAFs) displaying a proangiogenic phenotype

Melanoma-derived EVs display the potential to induce the differentiation of fibroblasts and/or ECs into CAFs. CAFs present in the TME can contribute to angiogenesis by directly altering the protein composition of TME but also by secretion matrix-degrading enzymes, for example. First of all, the transfer of lncRNA Gm26809 via melanoma-derived exosomes into normal fibroblasts induced their differentiation into CAFs [108]. Melanoma-derived exosomes carrying miR-155 have also been shown to induce CAF differentiation and downregulate the expression of the suppressor of cytokine signaling 1 (SOCS1) gene in recipient fibroblasts [109]. Downregulation of SOCS1 activated the Janus kinase 2/signal transducer and activator of transcription 3 (JAK2/STAT3) signaling pathway, resulting in proangiogenic switch and increased expression of VEGF, FGF2, and MMP-9 in recipient CAFs. Moreover, exosomes from miR-155-overexpressing B16 mouse melanoma cells significantly alleviated tube formation in 2D assay by MS-1 endothelial cell line and increased microvessel density in mice melanoma xenografts. In contrast, the downregulation of exosomal miR-155 brought opposite results, both *in vitro* and *in vivo*.

Also, Yeon et al. showed in a microfluidic 3D microvascular model that exosomes from melanoma cells stimulate the differentiation of ECs into CAFs [110]. This process was associated with increased expression of endothelial to mesenchymal transition-related genes, i.e., alpha smooth muscle actin (α-SMA), fibroblast-specific protein-1 (FSP-1), MMP-9, N-/VE-cadherins, vimentin, and TGF-β.

Finally, the most recent study analyzed whether EVs derived from melanoma cells cultured under hypoxia affect the proangiogenic properties of CAFs [111]. Hypoxia led to the enrichment of melanoma-derived EVs with HSP90/phosphorylated inhibitor of NF-κB kinase (IKK) (p-IKKα/β) complex. Subsequent EV-mediated transfer of HSP90/p-IKKα/β to CAFs activated the IKK/IκB/NF-κB signaling pathway and promoted CXCL1 expression and secretion in CAFs. In the same study, conditioned media from CAFs that underwent the described proangiogenic switch were added to HUVECs and increased their proliferation and tube formation in the 2D Matrigel assay. Moreover, in a xenograft murine model, mice inoculated with hypoxic EVs showed larger tumors than those inoculated with normoxic EVs. Moreover, blocking of EV-associated HSP90 with tanespimycin significantly reduced the size of xenografts. This proved the involvement of CAFs and HSP90/IKK/NF-κB/CXCL1 axis in the regulation of melanoma angiogenesis by EVs.

### Induction of proangiogenic properties of tumor-associated macrophages (TAMs)

The function of TAMs may also be regulated by melanoma-derived EVs. Due to the occurrence of their M1/M2 polarization, TAMs show varied functions in melanoma. Regarding angiogenesis, increased M2 polarization stimulates the formation of tumor vasculature. On the other hand, M1 TAMs have been shown to support the normalization of irregular vascular networks, which helps, for example, in the delivery of chemotherapeutics to tumor cells [112,113].

EVs are one of the mediators between melanoma cells and TAMs that may increase the latter’s proangiogenic properties. Back in 2016, Hood hypothesized that exosomes from melanoma cells increase the expression of granulocyte-macrophage colony-stimulating factor (GM-CSF) in ECs, and secreted GM-CSF increases the activity of HIF-2α in M2 TAMs [114]. HIF-2α further induces VEGFR-1 production, and activation of signaling pathways mediated by VEGF. Unfortunately, this hypothesis has not been confirmed experimentally. However, in a later study, Jarosz-Biej et al. [115] showed on melanoma tumor tissues that higher blood vessel density correlates positively with increased numbers of M1 rather than M2 TAMs.

Recently, Parikh et al. investigated the properties of melanosomes, a specific, melanin-transporting population of EVs derived from MNT-1 cells [116]. Unlike other EVs that are utilized by the recipient cell, melanosomes remain intact and can be further transferred to another cell. The isolated MNT1-derived melanosomes were used to treat CAFs and were then re-isolated and used to treat macrophages. From the very beginning, they carried AKT1, which stimulated mTOR-dependent VEGF secretion by macrophages, promoting angiogenesis in the murine model. Furthermore, the same study used human melanoma specimens and positively correlated macrophages histologically co-localized with AKT1 with more progressive disease. Moreover, samples from patients unresponsive to immunotherapy were enriched in macrophages expressing melanosome markers.

### Role of melanoma-derived EVs in lymphangiogenesis

Besides classical angiogenesis, melanoma-derived EVs regulate lymphatic vessel formation, as first shown in premetastatic niches in mice [117]. The observed effect was attributed to the nerve growth factor receptor (NGFR) transferred via EVs and activated ERK and NF-κB signaling in recipient lymphatic ECs. Consistently, EVs not expressing NGFR reduced the number of lymph node metastases and improved the survival of B16 mice. Moreover, lymphatic ECs were shown to overexpress intracellular adhesion molecules (ICAM-1) after EV treatment. Overexpression of ICAM-1 contributed to increased lymphangiogenesis and adhesion of melanoma cells.

**Figure 4:**
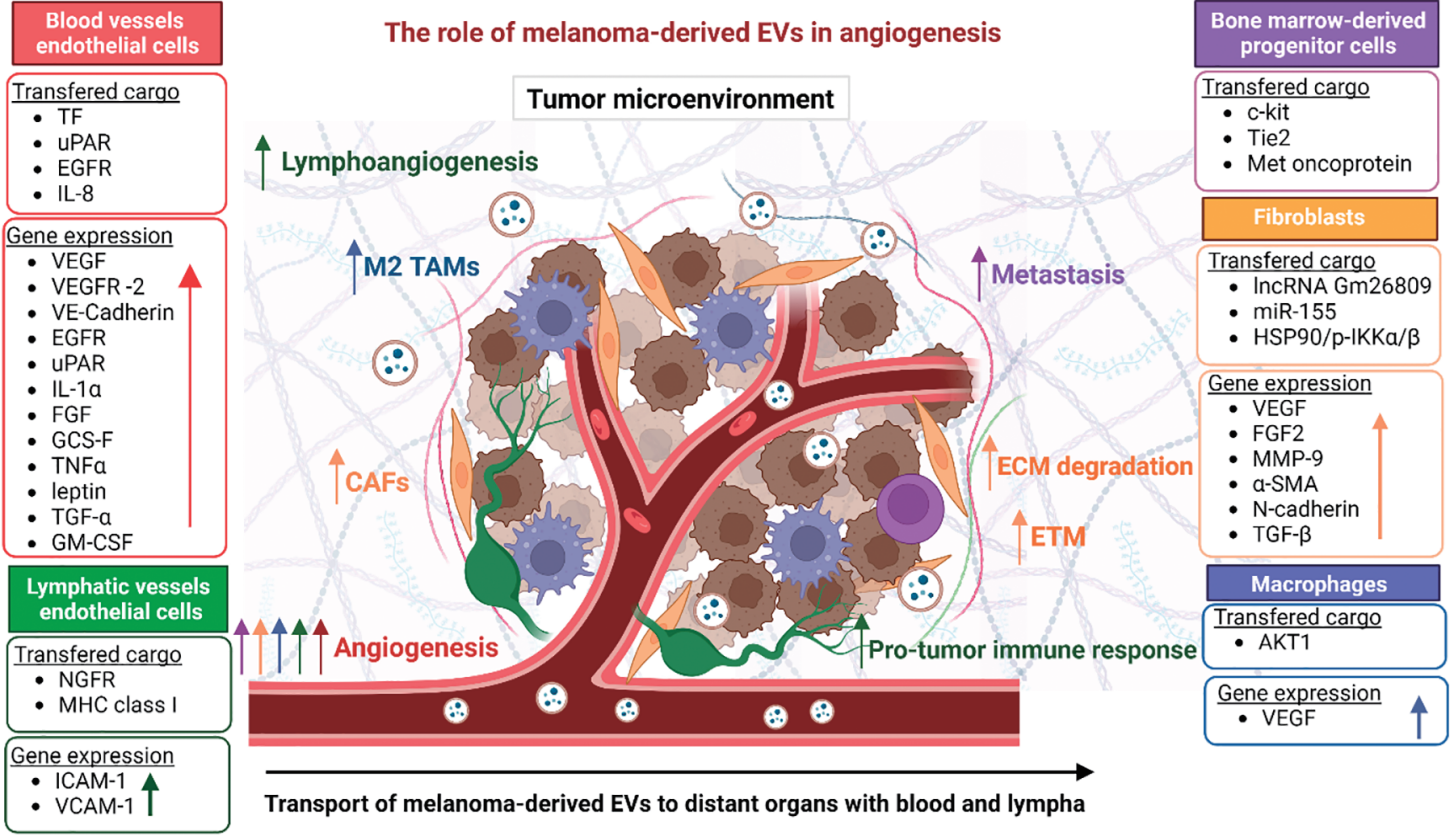
Summary of the role of melanoma-derived EVs in angiogenesis by affecting cells present in the TME. Cell types and their function are color-coded. The melanoma microenvironment consists of several types of cells, including immune system cells (TAMs, neutrophils, T cells) and ECs. These cells cooperate in multiple processes involved in melanoma progression, one of which is angiogenesis. The coexistence of cells forms paracrine loops regulating ECM remodeling, lymphoangiogenesis, and importantly the formation of new blood vessels and their maturation. The figure was prepared under the license in BioRender: Scientific Image and Illustration Software.

Another study also found interactions between melanoma-derived EVs and lymphatic EC, this time mediated by vascular cell adhesion molecule 1 (VCAM-1) [118]. EVs increased lymphatic ECs proliferation and lymph node remodeling. Moreover, EVs transferred various melanoma antigens and MHC-1 molecules, and their subsequent presentation by lymphatic ECs led to cytotoxic T cells apoptotic death and subsequent inhibition of the immune response. The role of melanoma-derived EVs in angiogenesis is summarized in Fig. 4.

## Clinical Potential of EVs in Antiangiogenic Therapies

Angiogenesis is undoubtedly a key step in melanoma progression, enabling faster tumor growth and local/distant metastases. Unfortunately, many diagnoses are made after the disease has reached this point, hindering the effectiveness of common therapeutic approaches. For these reasons, the majority of melanoma treatment strategies involve antiangiogenic drugs that target VEGF, VEGFR, FGFR, PDGFR, or integrins (summary in Table 1).

**Table 1:** Overview of antiangiogenic therapies in melanoma

Type of drug	Drug	Trade name	Target	Effect	Literature
Monoclonal antibodies	Bevacizumab	Avastin™	VEGF	Inhibition of VEGF-A binding to its receptors; decrease of ECs growth and vessel formation; can be administered in combination with paclitaxel and carboplatin;	[119,120]
	Aflibercept	EYLEA™, Zaltrap™ (ziv Aflibercept™)	VEGF/PlGF	decoy receptor for VEGF-A and PlGF with greater affinity than their natural receptors; inhibition of VEGF binding to VEGFR1 and VEGFR2; can be combined with pembrolizumab and IL-2;	[121–123]
	Etaracizumab	Abegrin™	αvβ3 integrin	Binding to αvβ3 integrin and inhibiting its function;	[124–126]
	Ramucirumab	Cyramza™	VEGFR-2	Inhibition of VEGF binding by binding to VEGFR-2 receptor; inhibition of angiogenesis;	[127]
	Ontuxizumab	Ontuxizumab™	Endosialin	Binds to endosialin (CD248/TEM1); inhibition of angiogenesis and tumor growth;	[128]
Tyrosine kinase inhibitors	Axtinib	Inlyta™	c-SRC, Kit, and RET, VEGFR-1/2,	Inhibition of VEGF signaling pathway;	[129–131]
	Imatinib	Imatinib™	PDGFR, c-kit, v-Abl	Inhibition of PDGF signaling pathway;	[132,133]
	Lenvatinib	Lenvima™, Lenvatinib™	VEGFR, FGFR	Inhibition of VEGF and FGF signaling pathways; can be combined with PD-L1 inhibitors;	[134–136]
	Pazopanib	Pazopanib™, Votrient™	c-kit, PDGFR-α/β, VEGFR-1/2/3	Inhibition of VEGFR1/2/3, PDGFR-α/β pathways;	[137,138]
	Sorafenib	Sorafenib™, Nexavar™	c-kit, FGFR-1, PDGFR-1, VEGFR	Inhibition of PDGFR-1, VEGFR and FGFR-1 pathways;	[139–141]
	Sunitinib	Sunitinib™, Krka™, Sutent™, Sutinib™	VEGFR	Inhibition of VEGFR pathway;	[142,143]
	Vatalanib		PDGFR-β, VEGFR-1/2/3	Inhibition of VEGFR and PDGFR-β pathways.	[144]

Although a modest improvement in overall survival has been observed following the administration of antiangiogenic therapies, there are still unresponsive patients who have developed resistance over time. EVs likely contribute to the failure of antiangiogenic therapies. In non-melanoma cancers, such as breast, colorectal, and renal cancers or glioblastoma, EVs have been shown to transfer different variants of VEGF with a much lower affinity for antiangiogenic drugs (mainly bevacizumab) than soluble VEGF in plasma. On the other hand, VEGF variants transferred via EVs show higher affinity for VEGFRs on ECs, which may represent a detour used by tumor cells to sustain angiogenesis [145].

However, there is another side to the EV story. The specific properties of EVs can be used to improve the effectiveness of antiangiogenic therapies in melanoma. First of all, abrogation of proangiogenic EV release in combination with antiangiogenic drugs could restore sensitivity to, for example, drugs targeting the VEGF/VEGFR axis. Known inhibitors of EV release/uptake by recipient cells are shown in Table 2 and have been thoroughly reviewed [146]. Unfortunately, no studies or clinical trials have been conducted on the combined use of EV inhibitors and anti-angiogenic drugs. However, recently, EVs in the plasma of healthy individuals were shown to transfer mostly anti-angiogenic proteins which suppress the angiogenic properties of recipient ECs [147]. On the other hand, EVs from patients with head and neck cancer increased migration and proliferation of ECs and their tube-forming potential. This suggests that targeting EV release may be an indirect way to abrogate tumor angiogenesis.

**Table 2:** Inhibitors of EV release or uptake

Action	Inhibitor	Literature
Inhibition of exosome formation	DPTIP (2,6-dimethoxy-4–(5-phenyl-4-thiophen-2-yl-1H-imidazole-2-yl)-phenol)	[154]
	Glyburide	[146]
	GW4869	[155]
	Imipramine	[156]
	Indomethacin	[157]
	Simvastatin	[158]
	Spiroepoxide	[159]
Inhibition of exosome formation and release	Ketoconazole	[160]
	Macitentan	[161]
	Manumycin A	[162]
	Sulfisoxazole	[163]
	Tipifarnib	[164]
Inhibition of exosome release	Calpeptin	[165]
	DMA (Dimethyl amiloride)	[166]
	Esomeprazole	[146]
	Ketotifen	[167]
	Lansoprazole	[168]
	Omeprazole	[146]
	Pantoprazole	[146]
Inhibition of exosome/ectosome release	Cannabidiol	[169]
Inhibition of ectosome release	Bisindolylmaleimide I	[170]
	Calpeptin	[171]
	Pantethine	[171]
	U0126	[172]
	Y-27632	[171]
Inhibition of exosome/ectosome uptake	Dynasore	[173]
	Methyl-β-cyclodextrin	[146]

Another thing to consider is the development of new drugs (antibodies, etc.) binding specifically to the different variants of proangiogenic factors expressed in EVs. Importantly, not only tumor-derived EVs shall be evaluated as potential targets for antiangiogenic therapies in melanoma. The function of other TEM cells such as ECs, CAFs, or macrophages, strongly depends on intercellular communication mediated by EVs. EVs, which, for example, induce polarization of M1 macrophages, should therefore be a focus of further research on tumor angiogenesis.

Moreover, EVs are biocompatible, non-toxic, and have high bioavailability. Because of these properties, they are used for drug delivery after being loaded with various drugs, including antiangiogenic agents used in melanoma treatment (Table 1). However, although EVs do not exhibit immunotoxicity, they can exhibit varying degrees of immunogenicity, which can potentially contribute to their increased clearance. The immunogenicity of EVs may be due to their surface molecular composition, as well as their internal cargo, cellular origin, dosage and infusion rate, etc. To mitigate this phenomenon, it is possible to use less differentiated cells as a source of EVs, modification EV cargo (removal of immune response-inducing molecules such as MHC-I or introduction of immune suppressors–complement regulator, PEG), reduction of dose and infusion rates, or use of smaller particles for drug delivery [148].

As mentioned above, no clinical trials strictly utilizing EVs in antiangiogenic therapy have been registered to date. However, the potential of various EV-based therapeutic strategies has been proven in several types of cancers and other diseases Almost 400 diagnostic or therapeutic trials utilizing EVs are registered on clinicaltrials.gov, of which more than 60 use EV therapy as the primary intervention, and articles have been already published extensively discussing this issue [149,150]. Currently, most trials focus on lung diseases, mainly due to the COVID-19 pandemic, although EVs are also used to treat acute respiratory distress syndrome and non-COVID-19 infections. Other important therapeutic applications of EVs include, among others, anti-rejection therapy post organ transplantation, gastroenterological diseases (inflammatory bowel disease, including Crohn’s and ulcerative colitis), hypercholesterolemia or nervous system conditions (Alzheimer’s, depression, neuralgia, or stroke) [149,150].

Finally, regenerative medicine is a field where EVs also show well-documented therapeutic potential. They are used for wound healing and regeneration (including burns, venous trophic lesions, etc.), treatments of bone defects and meniscal injuries, as well as muscle regeneration after myocardial infarction [149,150]. Importantly, tissue regeneration most often includes angiogenesis, so such trials indirectly indicate the potential of EV-based therapy in various conditions involving angiogenesis. For instance, there is a clinical trial using autologous plasma-derived exosomes for the management of most severe cutaneous ulcers (NCT02565264).

In cancer, however, enhanced angiogenesis is one of the factors contributing to cancer progression. The potential of EVs in antiangiogenic therapy has not yet been evaluated in clinical settings. Nevertheless, since the early 2000s, EV-oriented clinical trials have been developed for various cancers. The first trials used EVs as a vaccine to boost the anti-tumor immune response in colon cancer [151], non-small cell lung cancer [152] and melanoma [153]. Although they involved a small number of patients and showed unsatisfactory results, they clearly indicated the possibility and safety of therapeutic EV administration. In terms of ongoing trials, EV are currently being tested for the treatment of advanced hepatocellular carcinoma and liver metastasis of gastric and colorectal cancer (NCT05375604, EVs loaded with STAT6 antisense oligonucleotides), metastatic pancreatic cancer with a KRAS G12D mutation (NCT03608631, EVs loaded with KRAS G12D siRNA), and colon cancer (EVs loaded with curcumin).

None of the aforementioned trials was focused on angiogenesis, leaving the area unexplored for now. However, the data from basic research suggests that the antiangiogenic application of EVs may be a way to control or constrain the impact of tumor angiogenesis during cancer progression.

## Conclusions and Future Perspectives

**Figure 5:**
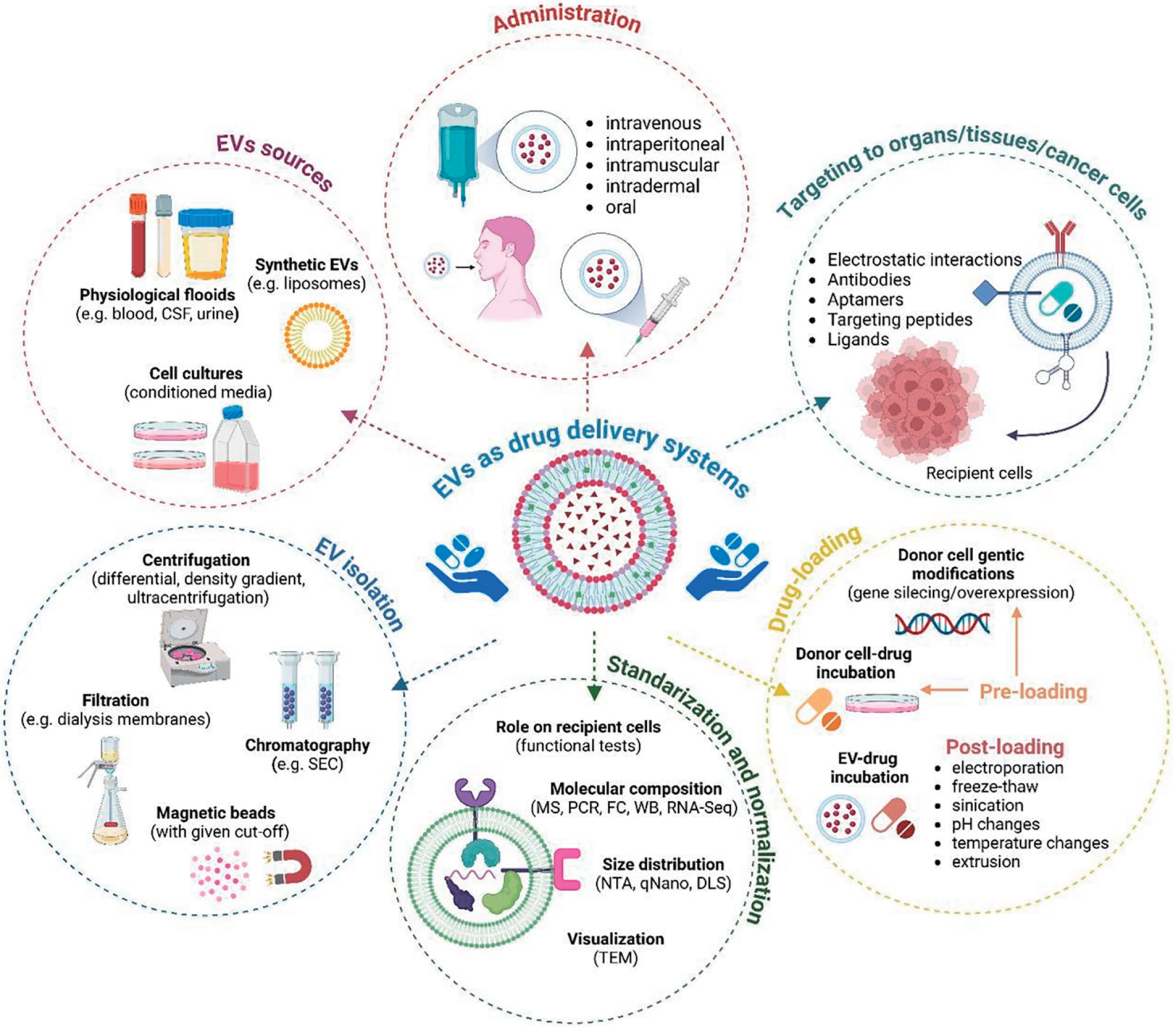
Various aspects of using EVs as drug delivery systems. The figure summarizes methods for isolating EVs from various sources, methods for standardizing and normalizing, methods for drug-loading and targeting them to recipient cells, and routes of administration. Used abbreviations: CSF–cerebrospinal fluid, DLS–dynamic light scattering, FC–flow cytometry, MS–mass spectrometry, NTA–nano tracking analysis, PCR–polymerase chain reaction, RNA seq–RNA sequencing, SEC–size exclusion chromatography, TEM–transmission electron microscopy, WB–western blotting. The figure was prepared under the license in BioRender: Scientific Image and Illustration Software.

Despite ongoing research in the EV field, there is a shortage of studies on melanoma-derived EVs in terms of their role and potential therapeutic application in antiangiogenic therapies. In particular, *in vivo* and clinical studies are lacking. Although the pro-/antiangiogenic action of EVs has been demonstrated in simpler models, there are still some limitations preventing the wider use of EVs in antiangiogenic therapies. For example, isolated/drug-loaded EVs must be strictly standardized in terms of their cellular source, concentration, purity, stability, etc. That should be followed by of the application of the most appropriate and efficient protocol for drug loading (Fig. 5). It is also necessary to ensure accurate biodistribution within the tumor vasculature, which involves providing therapeutic EVs with targeting molecules and assessing various routes of their administration. These issues make large-scale production of therapeutic EVs a major challenge. At that point, therapies based on artificial liposome-based EV-mimicking particles appear to be more within reach [174], as their production can be easily standardized and more widely commercialized. Another approach should include investigating which key proangiogenic components of EV cargo or signaling pathways activated by EVs in recipient cells during angiogenesis should be targeted by novel antiangiogenic therapies.

## Data Availability

Data sharing not applicable to this article as no datasets were generated or analyzed during the current study.
